# Vaccination Against COVID-19 Disease During Pregnancy

**DOI:** 10.15388/Amed.2021.29.1.11

**Published:** 2022-07-26

**Authors:** Austėja Voiniušytė, Miglė Černiauskaitė, Virginija Paliulytė, Rūta Einikytė, Diana Ramašauskaitė

**Affiliations:** Faculty of Medicine, Vilnius University, Vilnius, Lithuania; Center of Obstetrics and Gynaecology, Institute of Clinical Medicine, Faculty of Medicine, Vilnius, Lithuania; Center of Obstetrics and Gynaecology, Institute of Clinical Medicine, Faculty of Medicine, Vilnius University, Vilnius, Lithuania; Center of Obstetrics and Gynaecology, Institute of Clinical Medicine, Faculty of Medicine, Vilnius University, Vilnius, Lithuania; Center of Obstetrics and Gynaecology, Institute of Clinical Medicine, Faculty of Medicine, Vilnius University, Vilnius, Lithuania

**Keywords:** pregnancy, COVID-19 infection, vaccination, side effect

## Abstract

**Background.:**

The effect of COVID-19 disease during pregnancy is still under investigation, however scientific studies have shown that pregnant women with COVID-19 infection are at increased risk for severe illness or complications [1]. Risk factors for severe disease and death in pregnancy include maternal age (especially ≥35 years), obesity, preexisting medical comorbidities (particularly hypertension and diabetes or more than one comorbidity), and being unvaccinated [2]. Many societies of obstetricians and gynecologists recommend that all pregnant patients undergo COVID-19 vaccination [1]. The aim of this study is to observe demographic characteristics, including education, place of residence and type of employment of women who chose to be vaccinated against COVID-19 in Lithuania, as well as to investigate any adverse reactions following the COVID-19 vaccine during pregnancy and compare the results to published scientific data.

**Materials and methods.:**

An online questionnaire for pregnant women primarily located in Lithuania who received at least one dose of COVID-19 vaccine before giving birth has been launched in July, 2021. Data were entered via *Google Forms* and analyzed using *Microsoft Excel* and *IBM SPSS Statistics*. Literature review was performed on *PubMed* and *Google Scholar* search engines on inclusion criteria: publication date 2019–2021, used keywords *pregnancy, COVID-19, vaccination, side effects*.

**Results.:**

Data were collected from 227 women vaccinated against COVID-19 during pregnancy. It was observed that the most chosen vaccine was Pfizer-BioNTech BNT162b2 (196 out of 227 individuals (86%)). More pregnant women confirmed having fever after the second dose compared to the first dose (p=0.006). In addition, injection site pain was the most common local side effect after both doses (98%) and more common after the first dose compared to the second (p=0.002). Regarding systemic reactogenicity more women experienced fatigue after the second dose comparing to the first dose (p=0.01). Furthermore, more women were unable to engage in daily activities after the second dose (p=0.03). All other symptoms did not differ after doses 1 and 2.

**Conclusions.:**

Overall findings of this study did not suggest any obvious safety signals among pregnant individuals who received COVID-19 vaccine and all the side effects were comparable to the general population. Completed literature review indicates that pregnant women vaccinated against COVID-19 experience the same side effects as individuals in general population and no specific postvaccination reactions among pregnant individuals are observed.

## Santrauka.

**Įvadas.:**

COVID-19 ligos poveikis nėštumui dar nėra visiškai ištirtas, tačiau iki šiol atlikti moksliniai tyrimai parodė, kad nėščiosioms COVID-19 ligos eiga dažniau būna sunki ir komplikuota nei ne nėščių reprodukcinio amžiaus moterų [1]. Sunkios ligos ir mirties nėštumo metu rizikos veiksniai yra vyresnis amžius (ypač ≥35 metų), nutukimas, gretutinės ligos (ypač hipertenzija ir diabetas arba daugiau nei viena gretutinė liga) ir nesiskiepijimas [2]. Daugelis akušerių ir ginekologų draugijų rekomenduoja visoms nėščiosioms pasiskiepyti nuo COVID-19 [1]. Šio tyrimo tikslas yra apžvelgti moterų, kurios pasiskiepijo nuo COVID-19 ligos Lietuvoje, demografinius rodiklius, įskaitant jų išsilavinimą, gyvenamąją vietą, darbo pobūdį, taip pat ištirti, koks šalutinis poveikis joms pasireiškė po vakcinacijos, ir palyginti gautus rezultatus su duomenimis, publikuojamais mokslinėje literatūroje.

**Medžiagos ir metodai.:**

2021 metų liepos mėnesį paruošėme internetinę apklausą nėščiosioms, gyvenančioms Lietuvoje, kurios prieš gimdymą pasiskiepijo bent viena vakcinos nuo COVID-19 ligos doze. Duomenys buvo renkami per „Google Forms“ ir analizuojami naudojant „Microsoft Excel“ ir „IBM SPSS Statistics“. Literatūros paieška buvo atlikta „PubMed“ ir „Google Scholar“ duomenų bazėse pagal šiuos kriterijus: publikacijos data 2019–2021 m., vartojami raktiniai žodžiai *nėštumas, COVID-19, vakcinacija, šalutinis poveikis*.

**Rezultatai.:**

Gauti 227 nėščiųjų, pasiskiepijusių nuo COVID-19 ligos, duomenys. Rezultatai parodė, kad dažniausiai nėščiosios rinkosi Pfizer-BioNTech BNT162b2 vakciną (196 iš 227 respondenčių (86 %)). Daugiau nėščiųjų karščiavo po antros vakcinos dozės (p = 0,006). Skausmas šalia dūrio ar dūrio vietoje buvo dažniausias šalutinis poveikis po abiejų vakcinos dozių ir dažnesnis po pirmos vakcinos dozės (p = 0,002). Lyginant sisteminius šalutinius poveikius, daugiau moterų jautė nuovargį po antros vakcinos dozės (p = 0,01). Be to, daugiau moterų negalėjo užsiimti kasdiene veikla po antros vakcinos dozės (p = 0,03). Visi kiti simptomai po pirmos ir antros vakcinos dozės reikšmingai nesiskyrė.

**Išvados.:**

Mūsų tyrimo rezultatai ir atlikta literatūros apžvalga parodė, kad moterys, pasiskiepijusios nuo COVID-19 ligos nėštumo metu, patiria tokį patį šalutinį poveikį kaip ir bendrosios populiacijos pasiskiepijusios moterys ir jokio išskirtinio šalutinio poveikio nėščiosioms nebuvo nustatyta.

## Introduction

During vaccination against COVID-19 infection, concerns regarding vaccine safety for pregnant women have been raised. Even though pregnant women with COVID-19 infection have the same symptoms as the general population, studies show that they are more likely resulting in admission to an intensive care unit, mechanical ventilation or extracorporeal membrane oxygenation (ECMO) [3, 4, 9]. According to scientific data, pregnant women with COVID-19 infection are at increased risk for severe illness or complications (e.g., thromboembolic complications, hypertensive conditions, preterm labor, cesarean section, or death during pregnancy), as compared with nonpregnant reproductive age women [4-6, 9], thus preventing critical COVID-19 infection is important for both mother and fetus. Risk factors for severe disease and death in pregnancy include maternal age (especially ≥35 years), obesity, preexisting medical comorbidities (particularly hypertension and diabetes or more than one comorbidity), and being unvaccinated [2]. In addition, scientific data indicates that pregnant women with COVID-19 infection are at higher morbidity and mortality rate [7]. Currently available scientific data and the results of vaccinated pregnant women indicate the same efficacy of the COVID 19 vaccine as in the general population and no direct or indirect adverse effects on the developing fetus or the course of pregnancy have been observed [6].

Many societies of obstetricians and gynecologists recommend that all pregnant patients undergo COVID-19 vaccination [1]. Up to date recommendations given by Lithuanian Society of Obstetricians and Gynecologists (LSOG) include that all women, who plan pregnancy, are pregnant or lactating should be vaccinated with mRNA vaccine against COVID-19 [8]. The American College of Obstetricians and Gynecologists (ACOG) also recommends that COVID-19 vaccines should not be withheld from pregnant individuals or lactating individuals [9].

By this study we are aiming to observe demographic characteristics, including education, place of residence and type of employment of women who chose to be vaccinated against COVID-19 infection. Furthermore, the objective of this research is to investigate any adverse reactions following the COVID-19 vaccine during pregnancy and whether they differ after dose 1 and dose 2, as well as to compare the results of our research with the data published in the scientific literature.

## Methods

In July 2021, we launched an online questionnaire for pregnant women primarily located in Lithuania who received at least one dose of COVID-19 vaccine before giving birth. Enrollment to the study was voluntary. 243 women of age 19 to 52 (average 30.7 [SD=3.9]) with known pregnancy status who received at least 1 dose of COVID-19 vaccine had filled out the survey. However, only 227 completed questionnaires containing the necessary information were selected for the final evaluation. The remaining questionnaires were incomplete or containing irrelevant information. Data were collected on COVID-19 vaccination uptake and doses received, vaccination type, gestational age at vaccination, vaccination perception, as well as self-reported demographics and maternal characteristics. Further data were collected on vaccination outcomes after the first and second doses. The criteria for classifying the severity of outcomes as mild, moderate, or severe were given to the participants. Data were entered via *Google Forms* and analyzed using *Microsoft Excel* and *IBM SPSS Statistics*.

Literature analysis was performed on *PubMed* and *Google Scholar* search engines on inclusion criteria: publication date 2019–2021, used keywords *pregnancy, COVID-19, vaccination, side effects*.

## Results

Data were available for 227 pregnant women of whom 157 confirmed receiving 2 doses (Table 1). The majority received the Pfizer-BioNTech BNT162b2 vaccine (196 out of 227 individuals [86%]), the others received Vaxzevria AZD1222 (8%), Moderna mRNA-1273 (4%) and Janssen JNJ-78436735 (2%) vaccines. Most participants resided in cities, were working from home or in the office, and had completed higher education.

**Figure 1.**
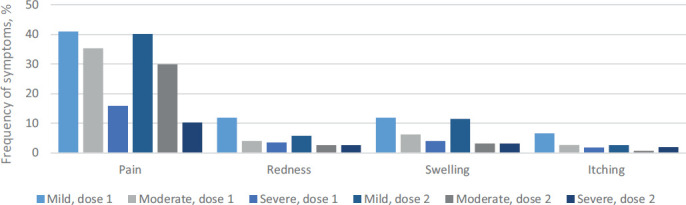
Postvaccination injection site reactions after dose 1 and 2

Among all respondents, 48 individuals reported having fever after the first dose, with the most common temperature being 37.0–38.5 °C (54%). Statistically significantly more pregnant women confirmed having fever after the second dose (p=0,006), with the most common temperature of 37.0–38.5 °C (84%). 213 women (93%) reported any postvaccination reactions at or near the injection site after the first dose including pain, swelling, redness and itching (Fig. 1). By contrast, 131 out of 157 individuals (83%) reported any of the forementioned reactions at or near the injection site after the second dose. Injection site pain was the most common local side effect after both doses (98%) and more common after the first dose compared to the second (p=0.002).

**Table 1.** Characteristics of the vaccinated pregnant women

Characteristics	N	%
Vaccine type		
Pfizer-BioNTech BNT162b2	196	86
Vaxzevria AZD1222	18	8
Moderna mRNR-1273	9	4
Janssen JNJ-7843675	4	2
Dose 1	227	100
Dose 2	157	69
Timing of first dose		
Before pregnancy	20	9
First trimester	79	35
Second trimester	90	40
Third trimester	38	17
Timing of second dose		
First trimester	50	32
Second trimester	68	43
Third trimester	37	24
After pregnancy	2	1
Number of pregnancies		
One	109	48
Two	89	39
Three	20	9
More than three	9	4
Number of deliveries		
One	127	56
Two	87	39
Three	9	4
More than three	4	1
Education		
Secondary	9	4
Incomplete higher	7	3
Higher (nonuniversity)	20	9
Higher (university)	191	84
Type of employment		
Work from home	75	33
Office work	64	28
Work with a close contact to other people	47	21
Manual labor	8	4
Health care	41	18
Not currently working	36	16
Residence		
City	203	89
Small town	17	8
Village	7	3
Information source about the possibility to get COVID-19 vaccine		
Internet	118	52
Friends and family	16	7
Health care institution	42	19
TV	21	9
Work	30	13
Incentive of vaccination		
My own decision	67	30
Friends and family	40	18
Health care workers	59	26
TV, internet, press	47	21
Work	14	6

**Figure 2.**
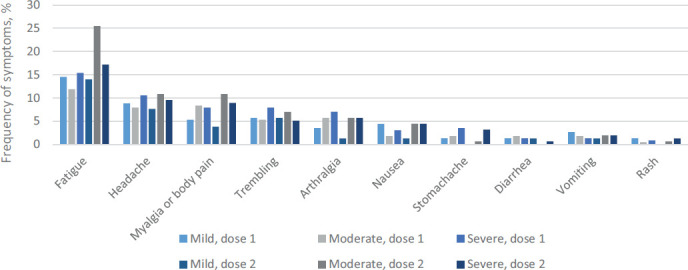
Postvaccination systemic reactions after dose 1 and 2

**Figure 3.**
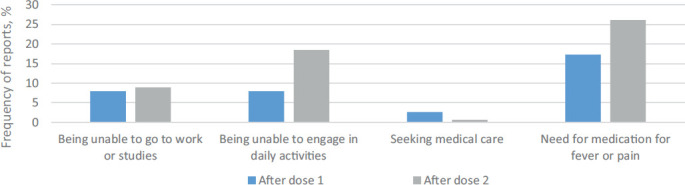
Postvaccination outcomes after dose 1 and 2

Further data were collected on general symptoms including fatigue, headache, myalgia or body pain, trembling, arthralgia, nausea, stomachache, diarrhea, vomiting and rash. 117 respondents (52%) reported having any of the aforementioned symptoms after the first dose and 109 respondents (69%) after the second dose (Fig. 2). Fatigue was the most frequently reported systemic side effect after both doses. Statistically significantly more women experienced fatigue after the second dose compared to the first dose (p=0.01). In addition, more women were unable to engage in daily activities after the second dose (p=0.03) (Fig. 3). All other symptoms did not differ after doses 1 and 2.

## Discussion

The reported side effects of COVID-19 vaccine in general population have mostly been mild or moderate and short-lasting [10]. In addition, most patients have recovered without the need of hospitalization. Centers for Disease Control and Prevention (USA) states that side effects from the vaccine are normal signs that body is building protection. Moreover, serious side effects that could cause long-term health problems are extremely unlikely following any vaccination, including COVID-19 vaccination [10, 11]. Our study limitations include uninvestigated duration of postvaccination reactions and outcomes.

The most common side effects of COVID-19 vaccine are local reactions which include pain at the injection site, redness, swelling, and systemic responses such as fever, fatigue, headache, myalgia, chills, diarrhea, and nausea [10, 11]. These reported patterns, with respect to both local and systemic adverse reactions, were similar to patterns observed among pregnant individuals in our survey-based research. We observed pregnant women vaccinated against COVID-19, who experienced local side effects including injection site pain, redness, swelling and itching, and systemic responses such as fatigue, headache, myalgia or body pain, trembling, arthralgia, nausea, stomachache, diarrhea, vomiting and rash. We found a similar trend for local reactions as for systemic reactions after both doses of vaccine. The most common symptoms observed were injection site pain and fatigue. Besides, in the study on short-term reactions among pregnant and lactating individuals in the first wave of the COVID-19 vaccine rollout performed in the USA in 2021 a similar pattern was found. Among all participants enrolled in the study the most common reported side effects were pain at the injection site and fatigue [5]. Furthermore, research on reactions and adverse events after the Pfizer-BioNTech BNT162b2 vaccine in general population released by Centers for Disease Control and Prevention (USA, 2021) indicates that the most frequent postvaccination symptoms in general population are also injection site pain and fatigue [12]. Moreover, in the v-safe pregnancy registry performed in the USA, 2021 it was found that local and systemic reactions that were reported to the v-safe surveillance system were similar among people who identified as pregnant and nonpregnant women. In the aforementioned study it was observed that pregnant individuals did not report having more severe reactions than nonpregnant individuals, except for nausea and vomiting, which were reported moderately more frequently after the second dose, however the overall reactogenicity profile was similar [6]. In addition, some of the clinical manifestations of COVID-19 overlap with symptoms of normal pregnancy (e.g., fatigue, shortness of breath, nausea or vomiting), which should be considered during evaluation of symptomatic pregnant women [2]. Therefore, these results indicate that pregnant women vaccinated against COVID-19 experience the same side effects as individuals in general population and no specific postvaccination reactions among pregnant individuals are observed.

Recently Centers for Disease Control and Prevention (USA) has released a new analysis of the latest data from the v-safe pregnancy registry on vaccination early in pregnancy. The research of nearly 2500 pregnant women who received a mRNA COVID-19 vaccine before 20 weeks of pregnancy did not find an increased risk of miscarriage [13]. Statistical data show that about 8 to 15 percent of clinically recognized pregnancies and around 30 percent of all pregnancies end in miscarriage [14]. In the forementioned study the rates of adverse pregnancy after receiving a COVID-19 vaccine were about 13 percent [13]. Although not directly comparable, calculated proportions of miscarriage in women vaccinated against COVID-19 are similar to the adverse pregnancy incidences in the general statistics. Moreover, an International Journal of Obstetrics and Gynaecology (BJOG) has published observed miscarriage rates after Pfizer-BioNTech BNT162b2 and Moderna mRNA-1273 vaccination. The results of the study show one miscarriage in the Pfizer-BioNTech BNT162b2 placebo group and no miscarriages in the vaccine group, along with one miscarriage in the Moderna mRNA-1273 placebo group and no miscarriages in the vaccine group [9]. In addition, another study findings on spontaneous abortion following COVID-19 vaccination during pregnancy in the USA show that among women with spontaneous abortion, the odds of COVID-19 vaccine exposure were not increased in the prior 28 days compared with women with ongoing pregnancies [15]. Thus, present results do not prove the increased risk of miscarriage after COVID-19 vaccine. Limitation of our study is uninvestigated miscarriages after receipt of COVID-19 vaccine. Further studies on adverse pregnancy outcomes associated with maternal COVID-19 vaccination in Lithuania are required.

## Conclusions

This study indicates that pregnant individuals in Lithuania prefer to be vaccinated with the Pfizer-BioNTech BNT162b2 vaccine during all trimesters of pregnancy over other vaccine types. Overall preliminary findings did not suggest any obvious safety signals among pregnant individuals who received COVID-19 vaccine and all the side effects were comparable to the general population. Completed literature analysis shows that pregnant individuals vaccinated against COVID-19 report the same side effects as individuals in general population and no specific postvaccination outcomes among pregnant women are observed.

## References

[1] Centers for Disease Control and Prevention. COVID-19 Vaccines While Pregnant or Breastfeeding. 2021 Dec 6. https://www.cdc.gov/coronavirus/2019-ncov/vaccines/recommendations/pregnancy.html.

[2] Berghella V, Hughes BL. COVID-19: Pregnancy issues and antenatal care. Literature review current through: Dec 2021. https://www.uptodate.com/contents/covid-19-pregnancy-issues-and-antenatal-care.

[3] Schwartz DA, Graham AL. Potential Maternal and Infant Outcomes from (Wuhan) Coronavirus 2019-nCoV Infecting Pregnant Women: Lessons from SARS, MERS, and Other Human Coronavirus Infections. Viruses. 2020 Feb 10;12(2):194. doi: 10.3390/v12020194. PMID: ; PMCID: . 32050635 PMC7077337

[4] Subbaraman N. Pregnancy and COVID: what the data say. Nature. 2021 Mar;591(7849):193-195. doi: 10.1038/d41586-021-00578-y. PMID: . 33692561

[5] Adhikari EH, Spong CY. COVID-19 Vaccination in Pregnant and Lactating Women. JAMA. 2021 Mar 16;325(11):1039-1040. doi: 10.1001/jama.2021.1658. PMID: . 33555297

[6] Shimabukuro TT, Kim SY, Myers TR, Moro PL, Oduyebo T, Panagiotakopoulos L, et al. Preliminary Findings of mRNA Covid-19 Vaccine Safety in Pregnant Persons. N Engl J Med. 2021 Jun 17;384(24):2273-2282. doi: 10.1056/NEJMoa2104983. Epub 2021 Apr 21. Erratum in: N Engl J Med. 2021 Oct 14;385(16):1536. PMID: ; PMCID: . 33882218 PMC8117969

[7] Villar J, Ariff S, Gunier RB, Thiruvengadam R, Rauch S, Kholin A, et al. Maternal and Neonatal Morbidity and Mortality Among Pregnant Women With and Without COVID-19 Infection: The INTERCOVID Multinational Cohort Study. JAMA Pediatr. 2021 Aug 1;175(8):817-826. doi: 10.1001/jamapediatrics.2021.1050. Erratum in: JAMA Pediatr. 2021 Nov 15;: PMID: ; PMCID: . 33885740 PMC8063132

[8] Lithuanian Society of Obstetricians and Gynecologists. Position of the Lithuanian Society of Obstetricians and Gynecologists regarding vaccination against COVID-19 infection for women, who plan pregnancy, are pregnant or lactating. 2021 Aug 6. https://lagd.lt/data/public/uploads/2021/08/2021-08-06_sam_lagd-pozicija-delplanuojanciu-pastoto-nesciuju-ir-zindanciu-moteru-skiepijimo-nuo-covid-19-infekcijos.pdf

[9] Joubert E, Kekeh AC, Amin CN. COVID-19 and novel mRNA vaccines in pregnancy: an updated literature review. BJOG. 2021 Oct 15:10.1111/1471-0528.16973. doi: 10.1111/1471-0528.16973. PMID: ; PMCID: . 34651406 PMC8652509

[10] World Health Organization. Coronavirus disease (COVID-19): VACCINES SAFETY. World Health Organization. 2021 Sep 21. https://www.who.int/news-room/q-a-detail/coronavirus-disease-(covid-19)-vaccines-safety.

[11] Centers for Disease Control and Prevention. Possible side effects after getting a covid-19 vaccine. Centers for Disease Control and Prevention. 2021 Nov 24. https://www.cdc.gov/coronavirus/2019-ncov/vaccines/expect/after.html.

[12] Centers for Disease Control and Prevention. Pfizer-biontech COVID-19 vaccine reactions & adverse events. Centers for Disease Control and Prevention. 2021 Nov 5. https://www.cdc.gov/vaccines/covid-19/info-by-product/pfizer/reactogenicity.html.

[13] Centers for Disease Control and Prevention. New CDC data: COVID-19 Vaccination safe for Pregnant people. Centers for Disease Control and Prevention. 2021 Aug 11. https://www.cdc.gov/media/releases/2021/s0811-vaccine-safe-pregnant.html.

[14] Linnakaari R, Helle N, Mentula M, Bloigu A, Gissler M, Heikinheimo O, et al. Trends in the incidence, rate and treatment of miscarriage-nationwide register-study in Finland, 1998-2016. Hum Reprod. 2019 Nov 1;34(11):2120-2128. doi: 10.1093/humrep/dez211. PMID: . 31747000

[15] Kharbanda EO, Haapala J, DeSilva M, Vazquez-Benitez G, Vesco KK, Naleway AL, et al. Spontaneous Abortion Following COVID-19 Vaccination During Pregnancy. JAMA. 2021 Oct 26;326(16):1629-1631. doi: 10.1001/jama.2021.15494. Erratum in: JAMA. 2021 Sep 10;:null. PMID: ; PMCID: . 34495304 PMC8427483

